# Awareness Level of Diabetes Mellitus Patients About Hypoglycemia Attacks and Management in Cluster 1, Riyadh, Saudi Arabia

**DOI:** 10.7759/cureus.100904

**Published:** 2026-01-06

**Authors:** Khalil Alduraibi, Saif M Alharbi, Mohammed G Alanazi, Huda I Khaleel, Maryam Alduraibi

**Affiliations:** 1 Family Medicine, King Saud Medical City, Riyadh, SAU; 2 Cardiology, King Saud Medical City, Riyadh, SAU; 3 Medicine, Imam Mohammad Ibn Saud Islamic University, Riyadh, SAU

**Keywords:** awareness, diabetes mellitus, hypoglycemia, patient education, self-management

## Abstract

Background

Diabetes mellitus is a common chronic condition that presents significant challenges for patients, particularly in managing hypoglycemia. The frequency of hypoglycemic episodes can significantly impact patients' quality of life and long-term health outcomes.

Aim

This study aimed to assess the awareness and management of hypoglycemia among people living with diabetes in Cluster 1, Riyadh, Saudi Arabia, focusing on their knowledge, practices, and preparedness for managing hypoglycemia.

Methods

This cross-sectional study involved 299 patients with diabetes from healthcare centers in Cluster 1, Riyadh, Saudi Arabia, in the period between February 1, 2025, and August 20, 2025. Data were collected through a structured survey that assessed demographic characteristics, knowledge of hypoglycemia, blood glucose monitoring practices, and preparedness for hypoglycemic episodes. The study also explored sources of information, frequency of healthcare consultations, and confidence in managing low blood sugar. Descriptive and statistical analyses were used to evaluate relationships between variables such as diabetes type, experience with hypoglycemia, and healthcare consultation frequency.

Results

The study included 299 patients with diabetes, with a higher proportion of male patients (187; 62.5%) compared to female patients (112; 37.5%). Age distribution was varied, with 63 (21.1%) participants aged 18-30 years, 63 (21.1%) aged 31-45 years, 104 (34.8%) aged 46-60 years, and 69 (23.1%) over 60 years of age. Type 2 diabetes was the most common diagnosis (62.5%), followed by Type 1 diabetes (n=76; 25.4%). The majority of participants exhibited poor knowledge regarding hypoglycemia, with 217 (72.6%) patients classified as having low awareness. Only 82 (27.4%) demonstrated good knowledge of hypoglycemia management.

Conclusions

This study reveals a low level of awareness and management of hypoglycemia among diabetic patients in Riyadh. There is a clear need for targeted education programs that address both the prevention and management of low blood sugar.

## Introduction

Diabetes mellitus is a chronic metabolic disorder characterized by hyperglycemia resulting from defects in insulin secretion, insulin action, or both [1]. The persistent hyperglycemia of diabetes is associated with long-term damage, dysfunction, and failure of various organs, especially the eyes, kidneys, nerves, heart, and blood vessels [2,3]. The disease is categorized into several types, with type 1 diabetes caused by an autoimmune-mediated destruction of pancreatic beta-cells leading to absolute insulin deficiency, and type 2 diabetes arising from a combination of insulin resistance and an inadequate compensatory insulin secretory response [4,5]. Other forms include gestational diabetes and specific types due to other causes [6]. Management focuses on glycemic control through lifestyle modifications, pharmacotherapy (including insulin and oral hypoglycemic agents), and regular monitoring to prevent acute complications and reduce the risk of long-term vascular complications [7,8].

 In Saudi Arabia, the prevalence of diabetes among individuals aged 15 and older was higher in urban areas [9]. According to a study by Al-Nozha et al., 12% of men and 14% of women in urban areas were affected by diabetes, while in rural areas, the rates were lower, with 7% of men and 7.7% of women affected [10]. Their study also highlights that 56% of diabetic patients were newly diagnosed and therefore unaware of their condition. In another study by Mirzaei et al., it was reported that 17% of individuals aged 30 and older were living with diabetes [11]. Al-Daghri et al.'s study highlights the growing burden of diabetes in Saudi Arabia and emphasizes that a substantial proportion of individuals with diabetes remain undiagnosed, supporting the statement that many patients are unaware of their condition [12].

Hypoglycemia is a serious complication of diabetes, mainly type 1, that can lead to heart problems and increased healthcare costs [13]. It also affects the quality of life, especially as hypoglycemic episodes become more frequent or severe [14]. Patients who are unaware of hypoglycemia are at a much higher risk of experiencing severe hypoglycemia, with the risk increasing by six to nine times [15]. About 25% of people with type 2 diabetes who have been on insulin for over five years experience severe hypoglycemia, a rate similar to that of people with type 1 diabetes diagnosed in the last five years [16]. The rate of recurring severe hypoglycemia (more than one episode) is 6.2% among those with good knowledge of hypoglycemia, compared to nearly 35% among those who are unaware [17]. The most common causes of severe hypoglycemia are insufficient food intake, physical activity, incorrect insulin doses, and reduced awareness of hypoglycemia [18].

The primary objective of our study was to assess the patients' awareness of hypoglycemia, including its symptoms, causes, and risk factors. Additionally, the study aims to evaluate their knowledge of effective management strategies for hypoglycemia and explore the connection between awareness levels and the frequency of hypoglycemic episodes. Given the high prevalence of diabetes and the significant health risks in Riyadh, this study aims to provide crucial insights that could enhance patient education and improve the management of hypoglycemia.

## Materials and methods

This was an observational cross-sectional study involving individuals aged 12 years and above, diagnosed with type 1 or type 2 diabetes mellitus. The study was reviewed and approved by the King Saud Medical City Institutional Review Board (proposal reference number: H1RI-27-Nov24-02).

Study population and sample size

Participants were recruited from local healthcare facilities within Cluster 1, Riyadh, Saudi Arabia, between February 1, 2025, and August 20, 2025, using a stratified random sampling approach to ensure representation across various demographic groups. The expected sample size was approximately 300 participants, based on the diabetes prevalence in the region and statistical power requirements.

Inclusion criteria included a confirmed diagnosis of type 1 or type 2 diabetes mellitus, age ≥12 years, residency in, or receipt of diabetes care within, Cluster 1, Riyadh, ability to provide informed consent, willingness to participate voluntarily, willingness and ability to complete the electronic or hard-copy survey on hypoglycemia awareness and management, and access to diabetes care through local healthcare centers or clinics within the cluster. Exclusion criteria included individuals with cognitive impairments, non-diabetic individuals, minors, pregnant women, and those with severe illnesses unrelated to diabetes.

Data collection

Data were collected using a combination of electronic and hardcopy self-reported surveys (see Appendices), which were distributed to patients attending diabetes care clinics. The survey questions assessed participants' knowledge of hypoglycemia, including its symptoms, causes, management strategies, and sources of information. The surveys were designed to capture both objective knowledge (e.g., specific symptoms of hypoglycemia) and self-reported awareness (e.g., patients' perceived understanding of hypoglycemia). Additional questions gathered demographic information, such as age, gender, educational level, and diabetes-related characteristics like the type of diabetes and duration of disease. Participants were also asked about their history of hypoglycemic episodes, healthcare consultations, and the sources from which they obtained information about hypoglycemia. Before the main study, a pilot study was conducted with a small sample of 15 patients to test the feasibility and clarity of the survey instrument. The results indicated that the questions were easily understood and appropriately addressed the study objectives. Minor adjustments were made to improve clarity and reduce the potential for response bias.

Data analysis

Data analysis was conducted using IBM SPSS Statistics for Windows, version 28 (Released 2021; IBM Corp., Armonk, United States). Descriptive statistics were used to summarize the demographic and clinical characteristics of the diabetic patients as well as their knowledge and awareness levels regarding hypoglycemia. Frequency distributions and percentages were calculated for categorical variables. The overall knowledge level of diabetic patients regarding hypoglycemia was assessed by assigning 1 point for each correct answer on a questionnaire. The total score was calculated, and participants who scored 60% or higher were classified as having good knowledge, while those scoring below 60% were classified as having poor knowledge. The inferential statistical method used to analyze the relationship between various demographic and clinical factors and knowledge levels was the Chi-square test (χ²). For small sample sizes, the Exact Probability Test was applied. A p-value less than 0.05 was considered statistically significant

## Results

A total of 299 patients were included in the study. Table 1 presents the bio-demographic characteristics of the participants. Considering age distribution, 21.1% (n=63) of participants were aged 18-30 years, another 21.1% (n=63) were in the 31-45 age range, 34.8% (n=104) were in the age group of 46-60 years, and 23.1% (n=69) were over 60 years of age. Regarding gender, a larger proportion of the sample was male (62.5%, n=187). Regarding educational level, the majority of patients had either a secondary (42.5%, n=127) or university-level education or higher (43.5%, n=130). When asked about the type of diabetes, most patients reported type 2 diabetes (62.5%, n=187), while 25.4% (n=76) had type 1 diabetes, and 12.0% (n=36) were unsure. As for the duration of diabetes, 12.4% (n=37) of patients have had diabetes for less than a year, 29.4% (n=88) for one to five years, 30.1% (n=90) for 6-10 years, and 28.1% (n=84) for more than 10 years. Regarding medications, 44.5% (n=133) of patients take oral hypoglycemics, 22.1% (n=66) use insulin injections, 25.1% (n=75) use both oral medications and insulin, and 8.4% (n=25) take no medication. Exactly 37.1% (n=111) of patients reported having hypoglycemic attacks. Only 7.4% (n=22) of patients had been hospitalized due to severe low blood sugar.

**Table 1:** Bio-demographic characteristics of study participants (N=299)

Characteristics	Frequency	Percentage
Age (years)		
18-30	63	21.1%
31-45	63	21.1%
46-60	104	34.8%
> 60	69	23.1%
Sex		
Male	187	62.5%
Female	112	37.5%
Educational level		
Below secondary education	42	14.0%
Secondary education	127	42.5%
University or above	130	43.5%
Type of diabetes mellitus		
Type 1 diabetes mellitus	76	25.4%
Type 2 diabetes mellitus	187	62.5%
Not sure	36	12.0%
Duration of diabetes mellitus		
< 1 year	37	12.4%
1-5 years	88	29.4%
6-10 years	90	30.1%
> 10 years	84	28.1%
Medications taken		
Oral hypoglycemics	133	44.5%
Insulin injections	66	22.1%
Both of them	75	25.1%
Nothing	25	8.4%
Low blood sugar attack		
Yes	111	37.1%
No	155	51.8%
Not sure	33	11.0%
Hospitalization due to severe low blood sugar		
Yes	22	7.4%
No	277	92.6%

Regarding patients' self-assessment of their knowledge of the symptoms of low blood sugar, the majority rated their knowledge as either intermediate (34.4%, n=103) or poor (26.1%, n=78), while fewer participants considered their knowledge good (25.1%, n=75) or excellent (14.4%, n=43) (Table 2). When asked about the symptoms of hypoglycemia, the most commonly recognized symptoms included shivering (69.1%, n=206), sweating (65.1%, n=194), and confusion (61.7%, n=184). Other symptoms like dizziness (56.7%, n=169), hunger (38.6%, n=115), tachycardia (31.9%, n=95), and weakness (20.1%, n=60) were less frequently mentioned. As for understanding the causes of hypoglycemia in diabetes, the majority of participants identified medication overdose (39.5%, n=118) and skipping meals (34.1%, n=102) as common triggers. A smaller percentage recognized excessive physical activity (10.0%, n=30) and stress (6.4%, n=19) as potential causes. Considering the immediate actions to take during hypoglycemia, the most widely acknowledged response was consuming something that contains sugar (81.6%, n=244), while fewer participants reported resting and waiting for symptoms to subside (10.4%, n=31) or seeking medical help (20.4%, n=61). About the preventive measures, 48.2% (n=144) of patients knew the importance of monitoring blood sugar levels, while 34.1% (n=102) recognized the need to eat regular snacks. Fewer patients mentioned adjusting insulin or other medication doses (10.0%, n=30) or managing physical activity (7.7%, n=23) as methods for preventing hypoglycemia.

**Table 2:** Knowledge and awareness of hypoglycemia symptoms, causes, and management among study participants (N=299)

Parameters	Frequency	Percentage
Knowledge of the symptoms of low blood sugar		
Poor	78	26.1%
Intermediate	103	34.4%
Good	75	25.1%
Excellent	43	14.4%
Symptoms of hypoglycemia		
Shivering	206	69.1%
Sweating	194	65.1%
Confusion	184	61.7%
Dizziness	169	56.7%
Hunger	115	38.6%
Tachycardia	95	31.9%
Weakness	60	20.1%
Causes of hypoglycemia in diabetes mellitus		
Overdose of medications	118	39.5%
Skipping meals	102	34.1%
Excessive physical activity	30	10.0%
Stress	19	6.4%
Alcohol intake	4	1.3%
I don't know	26	8.7%
First procedure that should be done when symptoms of low blood sugar appear		
Have something that contains sugar	244	81.6%
Rest and wait for symptoms to go away.	31	10.4%
Seek medical help	61	20.4%
Have more medications	5	1.7%
I don't know	16	5.4%
How to prevent hypoglycemia daily		
Eat regular snacks	102	34.1%
Monitoring blood sugar levels	144	48.2%
Adjust the doses of insulin or other medications	30	10.0%
Physical activity management	23	7.7%

**Figure 1:**
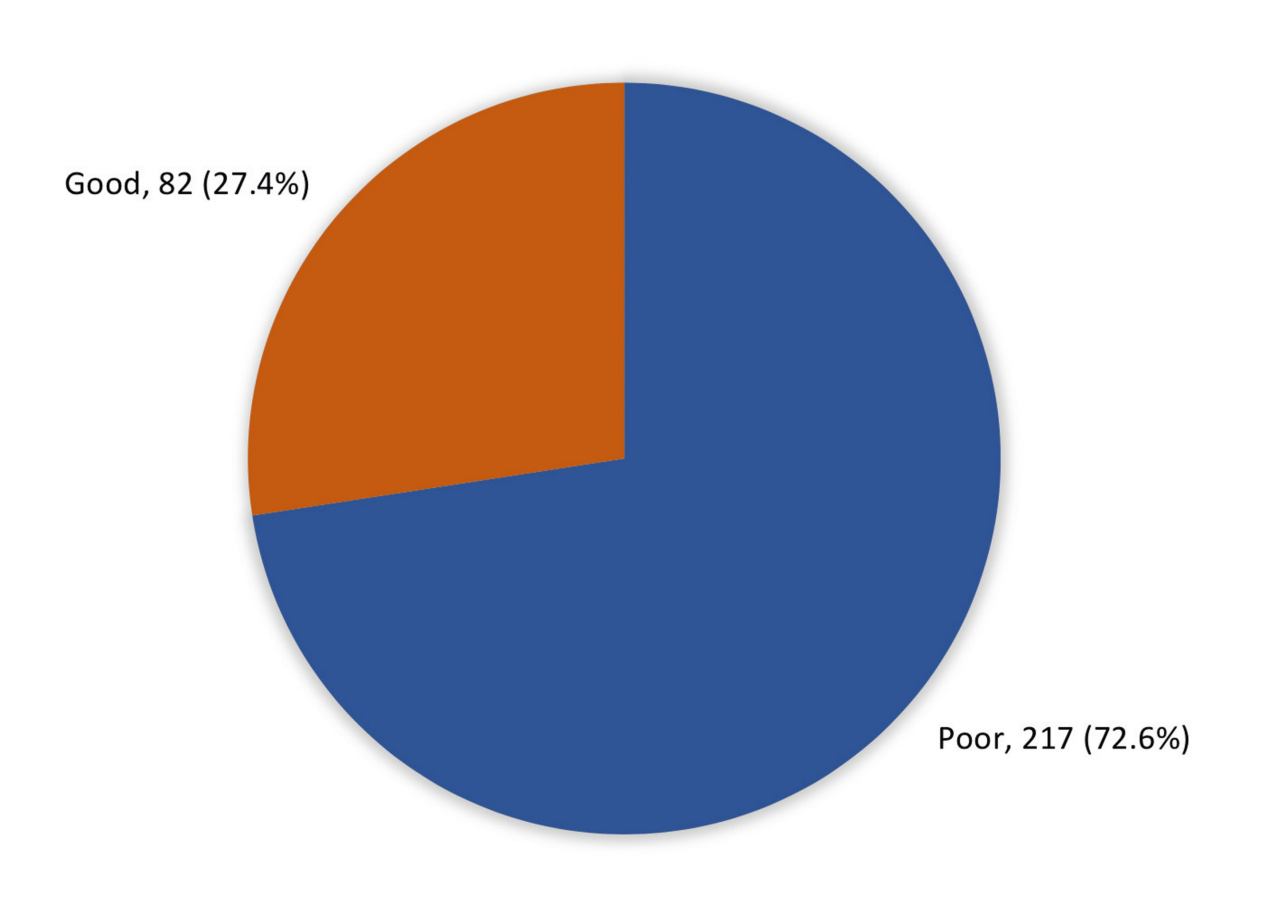
The overall knowledge and awareness of hypoglycemia among study participants (N=299)

**Figure 2:**
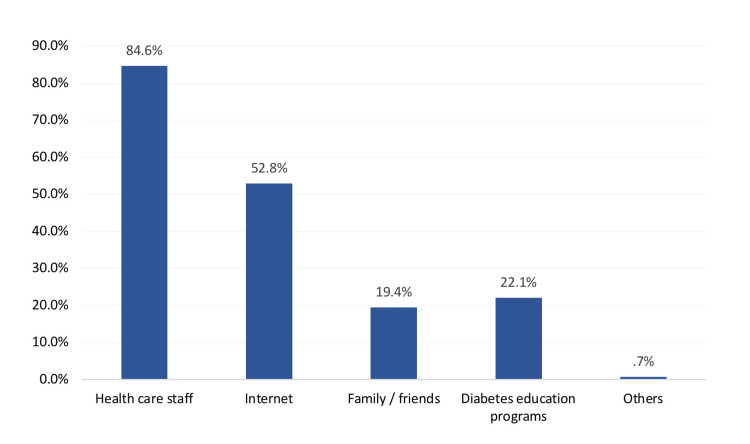
Source of information about hypoglycemia related to diabetes mellitus

Figure 1 shows the overall knowledge and awareness about diabetes-related hypoglycemia among the study participants. Concerning overall knowledge, a significant majority of participants (72.6%, n=217) had a poor knowledge level, while only 27.4% (n=82) had good knowledge. Regarding sources of information on hypoglycemia (Figure 2), healthcare staff were the most common source, with 84.6% (n=253) of participants relying on them for guidance. The internet also played a significant role (52.8%; 158). Family and friends were reported by 19.4% (n=58) of respondents, while diabetes education programs were identified by 22.1% (n=66) of participants. A very small proportion (0.7%, n=2) mentioned other sources.

Table 3 illustrates the practices of diabetic patients. Regarding the frequency of blood glucose monitoring, nearly a third of participants check their levels once a day (29.4%, n=88), while 27.4% (n=82) monitor their glucose many times a week. However, 8.4% (n=25) reported never checking their blood glucose, and 16.4% (n=49) check multiple times a day. Considering preparedness for low blood sugar, only 40.5% (n=121) of patients carry supplies such as glucose tablets or sweetened snacks for immediate treatment. When it comes to consulting healthcare providers about low blood sugar, a large proportion of participants (39.1%, n=117) consult their healthcare provider rarely (less than once a year), and 37.8% (n=113) do so only occasionally (every few months). A smaller percentage (5.4%, n=16) consult their healthcare provider regularly (monthly). Finally, 66.6% (n=199) of participants use a blood glucose monitoring system, such as continuous glucose meters or lancing devices, regularly. While this is a positive indicator of active management, a third of participants (33.4%, n=100) do not use such systems.

**Table 3:** Practices related to blood glucose monitoring and low blood sugar management among study participants (N=299)

Practice	Frequency	Percentage
Frequency of checking the blood glucose level		
Never	25	8.4%
Rarely	55	18.4%
Once a day	88	29.4%
Many times a week	82	27.4%
Many times a day	49	16.4%
Supplies carried for immediate treatment of low blood sugar (such as glucose tablets or sweetened snacks)		
Yes	121	40.5%
No	178	59.5%
Frequency of consulting healthcare provider about low blood sugar		
Never	53	17.7%
Rarely (< 1 time a year)	117	39.1%
Sometimes (every few months)	113	37.8%
Regularly (monthly)	16	5.4%
Use of a blood glucose monitoring system (such as continuous glucose meters or lancing devices) regularly		
Yes	199	66.6%
No	100	33.4%

Table 4 presents the levels of confidence among diabetic patients in Cluster 1, Riyadh, Saudi Arabia, in managing hypoglycemic attacks, as well as their exposure to education and need for further support. In terms of confidence, 43.1% (n=129) of patients reported feeling fairly confident in dealing with hypoglycemic attacks, while 12.7% (n=38) were very confident. However, 28.8% (n=86) were not confident. The majority of patients (56.5%, n=169) have not received formal education or training on how to manage low blood sugar. On the other hand, 43.5% (n=130) reported receiving formal education, which may correlate with the 12.7% of participants who feel very confident in managing low blood sugar. A large proportion of participants (73.6%, n=220) expressed a need for more information or support to better manage low blood sugar.

**Table 4:** Confidence in managing hypoglycemia and need for education among the study participants (N=299)

Parameters	Frequency	Percentage
Confidence in dealing with hypoglycemic attacks		
Very confident	38	12.7%
Fairly confident	129	43.1%
Not confident	86	28.8%
Don't know how to deal with it	46	15.4%
Formal education or training on how to manage low blood sugar		
Yes	130	43.5%
No	169	56.5%
Feel the need for more information or support to better manage low blood sugar		
Yes	220	73.6%
No	79	26.4%

Table 5 illustrates factors that are associated with diabetic patients' knowledge of hypoglycemia. The type of diabetes was found to be significant (p = 0.049), with type 1 diabetes patients exhibiting a higher proportion of good knowledge (32.9%) compared to Type 2 diabetes patients (28.3%). A history of low blood sugar attacks was strongly associated with better knowledge (p = 0.009). Patients who had experienced a low blood sugar attack had a higher percentage of good knowledge (36.9%) compared to those who had never experienced one (20.0%). Additionally, the frequency of healthcare consultations on low blood sugar was another significant factor (p = 0.001). Patients who consulted their healthcare provider regularly about low blood sugar had the highest proportion of good knowledge, with 47.8% of those who visited every few months. In contrast, only 7.5% of those who never consulted a healthcare provider had good knowledge. Lastly, the source of information on hypoglycemia was a key factor (p = 0.001). Those who received information from healthcare staff had the highest level of good knowledge (29.2%), while those who relied on family or friends had a significantly lower knowledge level, with 84.5% of them having poor knowledge. On the other hand, factors such as age, gender, educational level, duration of diabetes, and hospitalization history were not found to be statistically significant (p > 0.05).

**Table 5:** Factors associated with knowledge level about hypoglycemia in study participants

Factors	Overall knowledge level				p-value
	Poor		Good		
	Frequency	Percentage	Frequency	Percentage	
Age (years)					.646
18-30	46	73.0%	17	27.0%	
31-45	44	69.8%	19	30.2%	
46-60	73	70.2%	31	29.8%	
> 60	54	78.3%	15	21.7%	
Gender					.320
Male	132	70.6%	55	29.4%	
Female	85	75.9%	27	24.1%	
Educational level					.981
Below secondary education	31	73.8%	11	26.2%	
Secondary education	92	72.4%	35	27.6%	
University or above	94	72.3%	36	27.7%	
Type of diabetes mellitus					.049*
Type 1	51	67.1%	25	32.9%	
Type 2	134	71.7%	53	28.3%	
Not sure	32	88.9%	4	11.1%	
Duration of diabetes mellitus (years)					.080
< 1	29	78.4%	8	21.6%	
1-5	71	80.7%	17	19.3%	
6-10	58	64.4%	32	35.6%	
> 10	59	70.2%	25	29.8%	
Previous incidence of a low blood sugar attack					.009*
Yes	70	63.1%	41	36.9%	
No	124	80.0%	31	20.0%	
Not sure	23	69.7%	10	30.3%	
Hospitalization due to severe low blood sugar					.608
Yes	17	77.3%	5	22.7%	
No	200	72.2%	77	27.8%	
Frequency of consultation with healthcare provider about low blood sugar					.001*^
Never	49	92.5%	4	7.5%	
Rarely (< 1 time a year)	94	80.3%	23	19.7%	
Sometimes (every few months)	59	52.2%	54	47.8%	
Regularly (monthly)	15	93.8%	1	6.3%	
Formal education or training on how to manage low blood sugar					.539
Yes	92	70.8%	38	29.2%	
No	125	74.0%	44	26.0%	
Source of information about diabetes mellitus-related hypoglycemia					.001*^
Health care staff	179	70.8%	74	29.2%	
Internet	98	62.0%	60	38.0%	
Family/friends	49	84.5%	9	15.5%	
Diabetes education programs	45	68.2%	21	31.8%	
Others	1	50.0%	1	50.0%	

## Discussion

The study sample consisted of 299 diabetic patients from Cluster 1, Riyadh, with a different demographic profile. Participants were distributed across various age groups, with the largest proportion aged 46-60 years. The gender distribution showed a higher number of male participants. In terms of education, most patients had at least a secondary or higher level of education. Type 2 diabetes was more prevalent than type 1, with a smaller percentage of participants unsure of their diabetes type. The duration of diabetes varied, with the majority having had the condition for 1-10 years. A mix of oral hypoglycemics, insulin injections, or both was the reported medication.

In this study, more than one-third of patients with diabetesreported experiencing hypoglycemic attacks, with a smaller percentage having been hospitalized due to severe low blood sugar. These findings reflect a moderate prevalence of hypoglycemic episodes compared to both local and international studies. In a study conducted in Saudi Arabia, Elshebiny et al. reported that the incidence of hypoglycemia among the total studied diabetic patients was 90 (22.5%) in a year [19]. Most diabetic patients were found to have excellent compliance with their medications (60%). On the other hand, they had less compliance with diet control (23.3%) and regular exercise (26.7%). The prevalence of reported hypoglycemia was higher among patients with type 1 diabetes mellitus (82.5%) compared to patients with type 2 diabetes mellitus (12.5%). Another local study found that more than half of patients (52%) reported at least one attack of hypoglycemia during Ramadan, (29%) of them had more than four attacks. About two-thirds of attacks (67%) occurred in the morning and evening, while less than one-fourth had hypoglycemia at night (17%) [20].

Internationally, studies have reported varying prevalence rates of hypoglycemic episodes among diabetes patients. For instance, a study in the United States found that nearly 25% of patients with type 2 diabetes on insulin therapy experienced hypoglycemic attacks [21], which is lower than the findings of the current study. This could be attributed to the common use of sulfonureas, especially Gliclazide, in Saudi Arabia, as it is more accepted by patients than using injectable options like insulin [22]. In Europe, research has indicated that up to 40% of patients with type 1 diabetes report frequent hypoglycemic episodes [23], which is somewhat consistent with the results from this study but higher than those seen in patients with type 2 diabetes. Moreover, a global review revealed that hypoglycemia is a more frequent complication in insulin-treated patients, with rates ranging from 20% to 50% depending on the population, treatment regimen, and country [24].

The relatively low hospitalization rate for severe hypoglycemia in this study is in line with findings from other studies, such as one conducted in Canada, where less than 10% of patients with hypoglycemia required hospitalization [25]. However, other studies have shown higher hospitalization rates, particularly in patients with poorly controlled diabetes or those who experience recurrent hypoglycemic events [26,27]. This indicates that while many patients experience mild hypoglycemic symptoms, the severe consequences, such as hospitalization, remain less common, but still have significant health risks for a subgroup of patients.

Considering hypoglycemia knowledge and awareness, a significant majority of patients rated their own understanding of hypoglycemic symptoms as intermediate to poor, a self-assessment corroborated by the objective measure that found most participants had an overall "poor" knowledge level. While autonomic symptoms like shivering, sweating, and confusion were reasonably well-recognized by a majority, knowledge of other key symptoms (e.g., hunger, tachycardia), important causes beyond missed meals and medication overdose (e.g., exercise, stress), and nuanced preventive strategies (e.g., medication adjustment) was markedly low. This knowledge deficit is a significant concern, as inadequate understanding of hypoglycemia is a well-documented risk factor for its increased frequency and severity [28]. The most encouraging finding is that healthcare staff were the primary source of information for most patients, reflecting their essential role as trusted educators. However, the relatively low utilization of structured diabetes education programs suggests an area for substantial improvement in care delivery. The high reliance on the internet also presents both an opportunity for disseminating accurate information and a risk of exposure to misinformation. These results are consistent with other regional studies that have identified knowledge gaps in hypoglycemia awareness among Saudi populations with diabetes, particularly concerning the identification of non-classical symptoms and the role of behavioral factors like exercise [29,30]. On the other hand, other studies showed good awareness levels among diabetic patients, like that done in Al-Jouf [31] and in Al-Ahsa [32]. The pattern of recognizing basic corrective actions (like consuming sugar) but lacking knowledge in prevention matches with international studies that argue for moving beyond basic advice to more comprehensive self-management education programs to effectively reduce hypoglycemic risk [33].

Considering practices, a significant portion of participants did regular blood glucose monitoring, with some checking their levels daily or several times a week. However, some patients did not monitor their glucose levels frequently, and a small proportion reported checking their levels multiple times a day. The preparedness for hypoglycemia is also lacking, as only a limited number of patients carry essential supplies like glucose tablets or snacks for emergencies. About healthcare consultations, many patients rarely or occasionally discuss low blood sugar with their healthcare providers. Despite this, a majority of participants actively use blood glucose monitoring systems, such as continuous glucose meters or lancing devices. However, the lack of consistent monitoring and preparedness points to areas where patient education and healthcare support can be improved, particularly regarding hypoglycemia awareness and emergency preparedness.

Study limitations

This study has several limitations that should be reported. First, the cross-sectional design had limited ability for causal inference between patients’ awareness levels and their actual experiences with hypoglycemia. Second, data were collected using self-reported questionnaires, which may introduce recall bias or participation bias, as some participants might have overestimated their knowledge or practices. Third, the study was conducted only in Cluster 1 in Riyadh, which may limit the generalizability of the findings to other regions of Saudi Arabia with different healthcare infrastructures or population characteristics. Finally, the study did not assess other potentially influential factors, such as socioeconomic status, type of diabetes education received, or psychological barriers to self-care, which could further explain variations in awareness and management behaviors. While our study focused primarily on type 1 and type 2 diabetes, we suggest that including categories such as maturity-onset diabetes of the young (MODY), latent autoimmune diabetes in adults (LADA), and gestational diabetes mellitus (GDM) could enrich the work as the variability in hypoglycemia risk among different diabetic subgroups may influence the generalizability of the findings, an area for future research.

## Conclusions

This study showed an unsatisfactory level of knowledge about hypoglycemia and management among diabetic patients in Riyadh. While many patients are familiar with the symptoms of low blood sugar, there’s a clear need for better understanding of the causes, prevention, and effective responses to hypoglycemic events. A substantial number of patients reported not feeling fully confident in managing hypoglycemia, and many hadn’t received formal education on how to handle these situations. Although some participants actively monitored their blood glucose levels, a lack of preparedness, such as not carrying glucose tablets or snacks, was observed. Based on that, healthcare providers must offer more personalized education, mainly for those who have experienced hypoglycemic episodes. By focusing on practical strategies for preventing and managing low blood sugar, such as regular monitoring and having the right supplies on hand, patients can gain greater confidence in their ability to manage their condition. Additionally, increasing the availability of diabetes education programs and encouraging patients to consult with healthcare professionals more frequently could help.

## Appendices

Survey questions

Section 1: Demographic Information

1.​Age:

12-30

31-45

46-60

61 and above

2. Gender:

Male

Female

3.​Type of Diabetes:

Type 1

Type 2

unsure 

4.​ Duration of Diabetes (in years):

Less than 1

1-5

6-10

More than 10

5.​ Educational Level:

No formal education

Primary school

Secondary school

University degree or higher

6.​ Do you take insulin or oral diabetes medication?

Insulin

Oral medication

Both

None

Section 2: Awareness of Hypoglycemia

7.​ Have you ever experienced a hypoglycemic attack (low blood sugar)?

Yes

No

Not sure

8. How would you rate your knowledge of the symptoms of hypoglycemia?

Excellent

Good

Fair

Poor

9.​ Which of the following do you think are symptoms of hypoglycemia? (Check all that apply)

Sweating

Shaking

Confusion

Dizziness

Hunger

Fast heartbeat

weakness

Other (please specify): ____

10.​ What do you believe can cause hypoglycemia in people with diabetes? (Check all that apply)

Skipping meals

Too much insulin or medication

Excessive physical activity

Drinking alcohol

stress

I don’t know

11.​ How often do you check your blood sugar levels?

Several times a day

Once a day

A few times a week

Rarely

Never

Section 3: Management of Hypoglycemia

12.​ What is the first action you take when you feel symptoms of hypoglycemia?

Eat something sugary

Rest and wait for the symptoms to pass

Take more insulin

Seek medical help

I don’t know

13.​ How confident are you in your ability to manage a hypoglycemic episode?

Very confident

Somewhat confident

Not confident

I don’t know how to manage it

14.​ How do you prevent hypoglycemia on a daily basis? (Select all that apply):

Regular meals/snacks

Monitoring blood sugar levels

Adjusting insulin/medication

Exercise management

15.​ Do you carry any items (such as glucose tablets or sugary snacks) for immediate treatment of hypoglycemia?

Yes

No

*Section 4: Sources of Information* 

16.​ Where do you get most of your information about managing hypoglycemia?

Healthcare providers (doctors, nurses)

Internet

Friends or family

Diabetes education programs

Other (please specify): ____

17.  How often do you consult your healthcare provider regarding hypoglycemia?

Regularly (at least once a month)

Occasionally (once every few months)

Rarely (less than once a year)

Never

18.​Have you ever received formal education or training on how to manage hypoglycemia?

Yes

No

Section 5: Additional Information

19.​ Do you feel you need more information or support to better manage hypoglycemia?

Yes

No

20.​ Have you ever been hospitalized due to severe hypoglycemia?

Yes

No

21.​ Do you use a blood glucose monitoring system (CGM or finger pricks) regularly?

Yes

No 
